# Identification and Characterization of Long Non-Coding RNAs: Implicating Insights into Their Regulatory Role in Kiwifruit Ripening and Softening during Low-Temperature Storage

**DOI:** 10.3390/plants12051070

**Published:** 2023-02-27

**Authors:** Ruilian Lai, Xiaopei Wu, Xin Feng, Minxia Gao, Yu Long, Rujian Wu, Chunzhen Cheng, Yiting Chen

**Affiliations:** 1 Fruit Research Institute, Fujian Academy of Agricultural Sciences, Fuzhou 350013, China; 2 College of Horticulture, Shanxi Agricultural University, Jinzhong 030801, China

**Keywords:** kiwifruit, fruit ripening and softening, long non-coding RNA, lncRNA-mRNA interaction, low-temperature storage

## Abstract

Long non-coding RNAs (lncRNAs) are crucial players regulating many biological processes in plants. However, limited knowledge is available regarding their roles in kiwifruit ripening and softening. In this study, using lncRNA-seq technology, 591 differentially expressed (DE) lncRNAs (DELs) and 3107 DE genes (DEGs) were identified from kiwifruit stored at 4 °C for 1, 2, and 3 weeks in comparison with non-treated control fruits. Of note, 645 DEGs were predicted to be targets of DELs (DEGTLs), including some DE protein-coding genes (such as β-amylase and pectinesterase). DEGTL-based GO enrichment analysis revealed that these genes were significantly enriched in cell wall modification and pectinesterase activity in 1 W vs. CK and 3 W vs. CK, which might be closely related to the fruit softening during low-temperature storage. Moreover, KEGG enrichment analysis revealed that DEGTLs were significantly associated with starch and sucrose metabolism. Our study revealed that lncRNAs play critical regulatory roles in kiwifruit ripening and softening under low-temperature storage, mainly by mediating the expression of starch and sucrose metabolism and cell wall modification related genes.

## 1. Introduction

Kiwifruit is among the most recently domesticated fruit crops. With its rise as a major economic crop, the research on regulation of kiwifruit storage has extensively increased. Kiwifruit is a climacteric fruit and exhibits an ethylene burst during ripening [1]. However, its softening process, ethylene production, and the climacteric characteristics are temporally separated, which is quite different from typical climacteric fruits such as banana and tomato. Kiwifruit ripening can be divided into three phases, i.e., slow softening phase (Phase I), rapid softening phase (Phase II), and ethylene-dependent softening phase (Phase III). In Phase I, the ripening process is initiated but its fruit firmness reduces slowly. Kiwifruit firmness is swiftly reduced to approximately 20% of the harvest value during Phase II. Climacteric burst occurs in Phase III, with the fruit undergoing respiratory climacteric, becoming soft enough for eating, and developing characteristic flavors and aromas [2]. The distinctive ripening and softening process of kiwifruit has attracted significant research attention, and significant advances have been made by conducting many experiments to clarify the underlying ripening regulatory mechanism from physiological, biochemical, molecular, and other perspectives. Physiological and biochemical studies have revealed that starch degradation/soluble solid accumulation [3], plant hormone metabolism [4], cell wall metabolism [5], carbohydrate metabolism [6], secondary metabolism [7], and volatile emission [8,9,10] vary remarkably during kiwifruit ripening. Moreover, the activity changes in many enzymes, including polygalacturonase (PG) [11], xyloglucan endotransglucosylase/hydrolase [12], β-amylase (BAM) [13], α-amylase (AMY) [14], α-glucosidase (GAL) [6], 1-aminocyclopropane-1-carboxylic acid oxidase (ACO) [15], lipoxygenase (LOX) [16], and sucrose-phosphate synthase [17], have also been confirmed to play key roles in kiwifruit ripening and softening.

Rapid progress has been made in elucidating the molecular mechanism underlying fruit ripening and softening. Many protein-coding genes (PCGs) and transcription factor genes (TFs), such as *PG* [18,19], *BAM* [20], *GAL* [21], pectin methylesterase (*PME*) [18], pectate lyase (*PL*) [22], *WRKY* [23], *NAM-ATAF1/2-CUC2* (*NAC*) [24,25], Homeobox [26], and zinc finger proteins (*ZFP*) [27], have been proven to be involved in the fruit ripening process of many plants. In kiwifruit, several PCGs, including *PG* [28,29], *PL* [22], *gretchen hagen 3* (*GH3*) [30], and *MADS-box* TFs [31], were also found to function in the regulation of fruit ripening and softening. High-throughput sequencing technology is a powerful tool that has been used for investigating the expression changes in transcripts during certain biological processes or under certain treatments. Using this technique, two distinct and independent ripening mechanisms, i.e., the ethylene-dependent ripening and the low-temperature-regulated ripening, were discovered in kiwifruit [32]. Genes and regulators involved in starch degradation, cell wall metabolism, hormone metabolism, fruit quality change, respiration rate regulation, stress response, and ester biosynthesis were identified to play roles in kiwifruit fruit ripening and softening [27,33,34]. Moreover, some small RNAs (sRNAs) that target PCGs or TFs regulating kiwifruit ripening and softening have been verified using sRNA-seq [35].

Long non-coding RNAs (lncRNAs) are involved in various biological processes/functions including RNA silencing, transcription and chromosome structure, mRNA stability and expression, and protein stability and transport [36,37]. With the rapid improvement of sequencing technology, numerous lncRNAs have recently been identified to play pivotal roles in many biological processes. In kiwifruit, the roles of lncRNAs have also been reported. Tang et al. [38] identified 7051 lncRNAs from *Actinidia chinensis* cv. ‘Hongyang’ fruits at different development and ripening stages. Zhao et al. [39] identified 149 differentially expressed lncRNAs (DELs) in *Wickerhamomyces anomalus* treated wounds in kiwifruit compared to non-treated controls, and found that these DELs might function in enhancing the kiwifruit resistance to *Penicillium expansum* and *Botrytis cinerea* by enhancing the expression of phytohormone-biosynthesis-related genes, and by regulating the expression of *TFs* (such as *WRKY72*, *WRKY53* and *JUB1AP2*) mediating the expression of defense-related and secondary metabolites’ biosynthesis-related genes. In one of our previous studies [14], the predicted target genes of DELs identified in ABA-treated kiwifruit were found to be significantly enriched in plant hormone transduction, flavonoid biosynthesis, and some other pathways.

Temperature is a key factor affecting kiwifruit storage [3,40]. Extensive ripening of kiwifruit in cold storage occurs in the absence of any detectable ethylene [13,32]. The regulatory role of lncRNAs in ethylene-mediated kiwifruit ripening and softening has been studied [41]. Until now, however, the function of lncRNAs in the ripening and softening of low-temperature-stored kiwifruit is still unclear. In this study, to explore the regulatory roles of lncRNAs during this process, lncRNA-seq analysis was performed for kiwifruit stored at 4 °C for 0, 1, 2, and 3 weeks. Our study will be helpful in clarifying the regulatory roles of lncRNAs in kiwifruit during low-temperature storage.

## 2. Results

### 2.1. Physiological Properties Comparisons of Kiwifruit Stored at 4 °C for 0, 1, 2, and 3 Weeks

**Figure 1:**
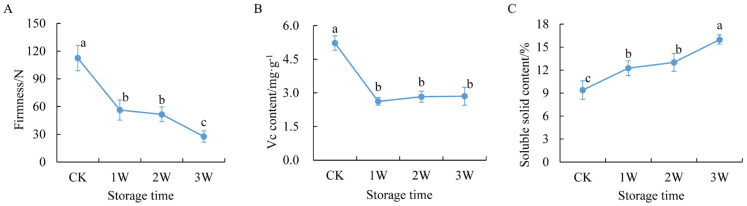
Physiological properties of kiwifruit during low temperature storage. (**A**–**C**) represent the change in firmness, vitamin C content, and soluble solid content during low-temperature storage, respectively. CK, 1 W, 2 W, and 3 W indicate that kiwifruit stored at 4 °C for 0, 1, 2, and 3 weeks, respectively. Different lowercases above curves represent a significant difference at *p*-value < 0.05 level.

During low-temperature storage, the firmness of most fruits gradually decreased (Figure 1). The firmness decreased significantly from approximately 112.42 N in the CK group to 56.23 N in the 1 W group, and further decreased from 51.57 N in the 2 W group to 27.62 N in the 3 W group (Figure 1A). The vitamin C (Vc) content in kiwifruit decreased rapidly from 5.22 mg·g^−1^ in the CK group to 2.62 mg·g^−1^ in the 1 W group. Then, the Vc content changed slightly during low-temperature storage (Figure 1B). However, the soluble solid content (SSC), a vital factor influencing kiwifruit flavor, increased from 9.39% in the CK group to 15.98% in the 3 W group during low-temperature storage (Figure 1C). 

### 2.2. LncRNA-Seq and Identification of LncRNAs

A total of 96,299,434, 95,968,050, 102,409,444, and 127,966,632 clean reads were obtained from the CK, 1 W, 2 W, and 3 W groups, respectively. The Q20 and Q30 values of the all libraries were >96% and >91%, respectively. The unbiased ratios of GC contents ranged from 45.40% to 49.70% (Table 1). In total, 75.13%, 78.26%, 75.29%, and 76.40% of the clean reads from the CK, 1 W, 2 W, and 3 W groups could be successfully mapped to the reference kiwifruit genome, while the uniquely mapped reads accounted for 70.40%, 74.37%, 69.34%, and 72.83%, respectively.

### 2.3. Expression Profiles of MRNAs

**Figure 2:**
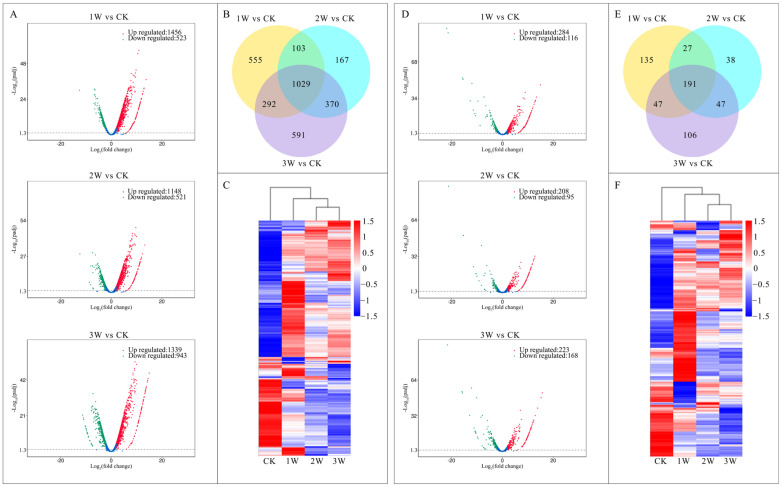
Expression profiles of DEGs and DELs in kiwifruit during low-temperature storage. (**A**–**C**) represent volcanic map, Venn diagram, and heatmap of DEGs, respectively. (**D**–**F**) represent volcanic map, Venn diagram, and heatmap of DELs, respectively. Each dot in the volcanic map represents a gene, the red, green, and blue color represents the expression of this gene is up-regulated, down-regulated, and not changed, respectively. Each column in the heatmap represents a treatment, and each row represents a gene, the red color indicates high expression and blue color indicates low expression of a gene in this treatment.

A total of 3107 differentially expressed genes (DEGs, Table S1), including 3017 known genes and 90 novel genes, were identified from low-temperature stored kiwifruit compared with the non-treated controls using the DEGseq package with corrected *p*-value < 0.05 and |log_2_^fold change (FC)^| ≥ 2.0 as criteria. Compared with the CK group, 1979 (1456 upregulated and 523 downregulated), 1669 (1148 upregulated and 521 downregulated), and 2282 (1339 upregulated and 943 downregulated) transcripts were identified as DEGs in 1 W, 2 W, and 3 W groups (Figure 2A), respectively. Notably, 1029 DEGs, including 990 known genes and 39 novel genes, were commonly found in all the three comparisons. Moreover, 103, 292, and 370 DEGs were present in the comparisons of 1 W vs. CK and 2 W vs. CK, 1 W vs. CK and 3 W vs. CK, and 2 W vs. CK and 3 W vs. CK, respectively (Figure 2B). DEG-based clustering results revealed that the 2 W and 3 W groups had similar gene expression patterns, followed by the 1 W group, whereas the CK group was obviously distinguished from other groups (Figure 2C).

Among the 1029 common DEGs, 783 genes were upregulated while 226 genes were downregulated in all the three comparisons (Table S2). Compared with CK, after 1 W, 2 W and 3 W of low-temperature storage, several genes continuously and greatly upregulated (log_2_^FC^ > 10.0), including Acc13502 (Early light-induced protein 1), Acc23128 (Glucan endo-1,3-beta-glucosidase), Acc06784 (ABC transporter G family member 11), Acc05901 (Heavy metal-associated isoprenylated plant protein 21), and Acc14979 (WRKY transcription factor 6), while the expressions of some genes greatly decreased (log_2_^FC^ < −6.0), such as Acc16083 (Cinnamoyl-CoA reductase-like *SNL6*) and Acc27730 (Aspartic proteinase *PCS1*).

### 2.4. Expression Profiles of LncRNAs

A total of 591 differentially expressed lncRNAs (DELs, Table S3) were identified from low-temperature stored kiwifruit compared with the non-treated controls using the DEGseq package with corrected *p*-value < 0.05 and |log_2_^FC^| ≥ 2.0 as criteria. Compared with the CK group, 400 (284 upregulated and 116 downregulated), 303 (208 upregulated and 95 downregulated) and 391 (223 upregulated and 168 downregulated) transcripts were identified as DELs in 1 W, 2 W, and 3 W groups (Figure 2D), respectively. Notably, 191 DELs were commonly found in all the three comparisons. Among the 191 common DELs, 150 DELs were upregulated while 36 DELs were downregulated in all the three low-temperature stored groups (Table S4). Moreover, 27, 47, and 47 DELs were present in the comparisons of 1 W vs. CK and 2 W vs. CK, 1 W vs. CK and 3 W vs. CK, and 2 W vs. CK and 3 W vs. CK, respectively (Figure 2 E). DEL-based clustering results revealed that, similar to DEGs, the 2 W and 3 W groups had similar expression patterns, followed by the 1 W group, whereas the CK group was obviously distinguished from other groups (Figure 2F).

### 2.5. GO and KEGG Enrichment Analyses of DEGs

**Figure 3:**
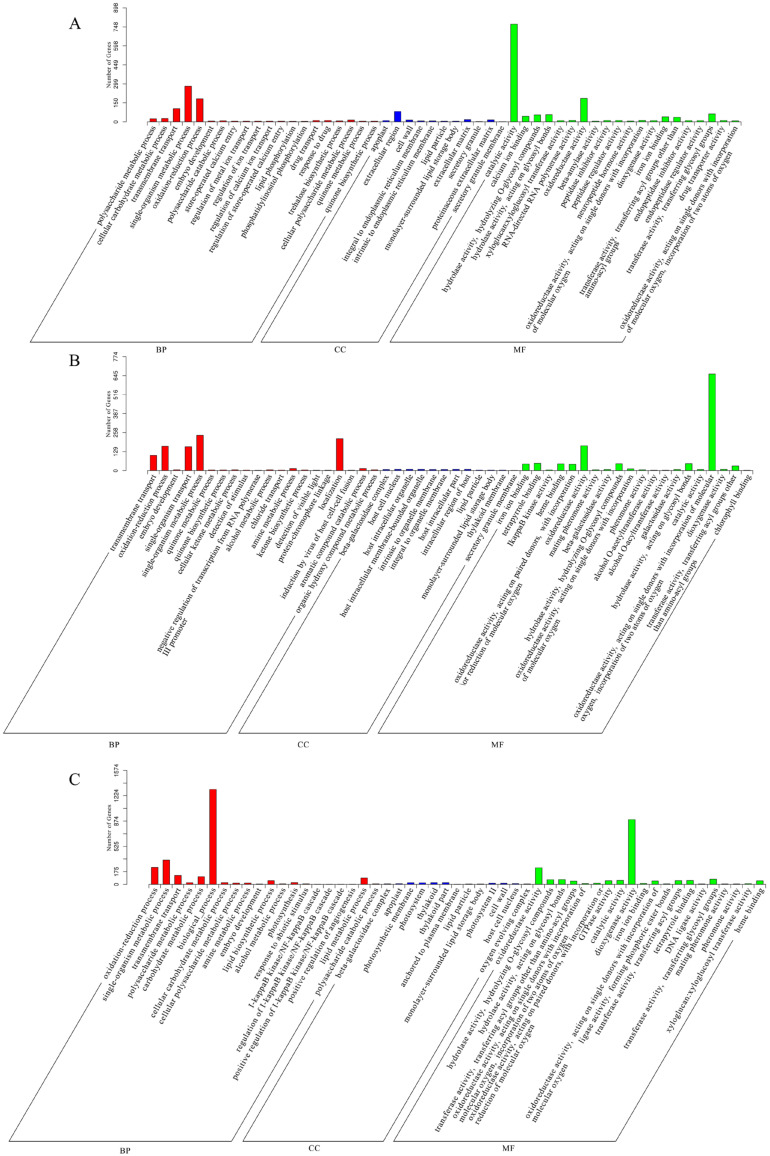
Top 20 significantly enriched GO terms (*p* < 0.05) for DEGs identified in low-temperature stored kiwifruit. BP: biological process; CC: cellular component; MF: molecular function. (**A**) Significantly enriched GO terms by DEGs in the comparison of 1 W vs. CK. (**B**) GO terms significantly enriched by DEGs in the comparison of 2 W vs. CK. (**C**) GO terms significantly enriched by DEGs in the comparison of 3 W vs. CK.

GO enrichment analysis was performed to illuminate the potential functions of DEGs. Many biological process (BP), molecular function (MF) and cellular component (CC) GO terms were significantly enriched in kiwifruit in the three comparisons (*p* < 0.05) (Table S5, Figure 3). Results showed that, there were 153, 140, and 171 GO terms significantly enriched in the 1 W vs. CK, 2 W vs. CK, and 3 W vs. CK, respectively. In addition, the DEGs identified in three groups were all found to be significantly enriched in 50 GO terms, including ‘transmembrane transport’, ‘oxidation-reduction process’, ‘single-organism metabolic process’, ‘polysaccharide catabolic process’, ‘oxidoreductase activity’, ‘hydrolase activity, hydrolyzing O-glycosyl compounds’, ‘hydrolase activity, acting on glycosyl bonds’, ‘amylase activity’, ‘beta-amylase activity’, ‘lipid particle’, and ‘monolayer-surrounded lipid storage body’. Furthermore, the ‘polysaccharide metabolic process’, ‘glucan metabolic process’, ‘cell wall’, ‘carbohydrate metabolic process’, ‘lipid metabolic process’, and ‘transferase activity, transferring hexosyl groups’ GO terms were significantly enriched by DEGs in both 1 W and 3 W groups.

**Figure 4:**
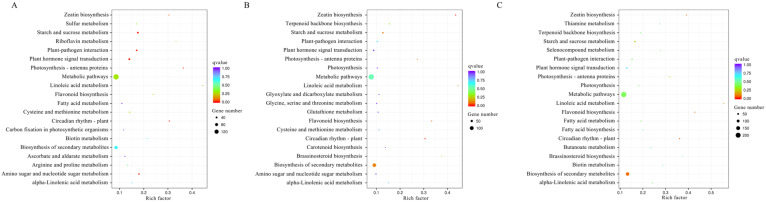
KEGG pathway enrichment analysis of DEGs in low-temperature stored kiwifruit. (**A**) Top 20 pathways enriched by DEGs in the comparison of 1 W vs. CK. (**B**) Top 20 pathways enriched by DEGs in the comparison of 2 W vs. CK. (**C**) Top 20 pathways enriched by DEGs in the comparison of 3 W vs. CK.

KEGG enrichment analysis of DEGs was further performed (Table S6). Compared with the CK group, 101 KEGG pathways were identified in 1 W. Of these, 13 pathways were significantly enriched, including ‘Starch and sucrose metabolism’, ‘Plant-pathogen interaction’, ‘Circadian rhythm-plant’, ‘Amino sugar and nucleotide sugar metabolism’, ‘Plant hormone signal transduction’, ‘Photosynthesis-antenna proteins’, ‘Zeatin biosynthesis’, ‘Linoleic acid metabolism’, ‘Cysteine and methionine metabolism’, ‘Flavonoid biosynthesis’, ‘Metabolic pathways’, ‘Sulfur metabolism’, and ‘Riboflavin metabolism’ (Figure 4A).

After low-temperature storage for 2 W, DEGs were significantly enriched in nine pathways (*p* < 0.05), including ‘Zeatin biosynthesis’, ‘Circadian rhythm-plant’, ‘Flavonoid biosynthesis’, ‘Biosynthesis of secondary metabolites’, ‘Starch and sucrose metabolism’, ‘Linoleic acid metabolism’, ‘Photosynthesis-antenna proteins’, ‘Terpenoid backbone biosynthesis’, and ‘Brassinosteroid biosynthesis’ (Figure 4B).

In the 3 W vs. CK comparison, 110 pathways were enriched and 12 pathways were identified to be significantly enriched, including ‘Circadian rhythm-plant’, ‘Flavonoid biosynthesis’, ‘Zeatin biosynthesis’, ‘Biosynthesis of secondary metabolites’, ‘Linoleic acid metabolism’, ‘Starch and sucrose metabolism’, ‘Photosynthesis-antenna proteins’, ‘Fatty acid metabolism’, ‘alpha-Linolenic acid metabolism’, ‘Metabolic pathways’, ‘Photosynthesis’, and ‘Plant-pathogen interaction’ (Figure 4C).

Notably, ‘Zeatin biosynthesis’, ‘Circadian rhythm-plant’, ‘Flavonoid biosynthesis’, ‘Starch and sucrose metabolism’, ‘Linoleic acid metabolism’, and ‘Photosynthesis-antenna proteins’ pathways were found to be significantly enriched by DEGs in all the three comparisons.

### 2.6. GO and KEGG Enrichment Analyses of DEGTLs (DEGs That Were Predicted Target Genes of DELs)

**Figure 5:**
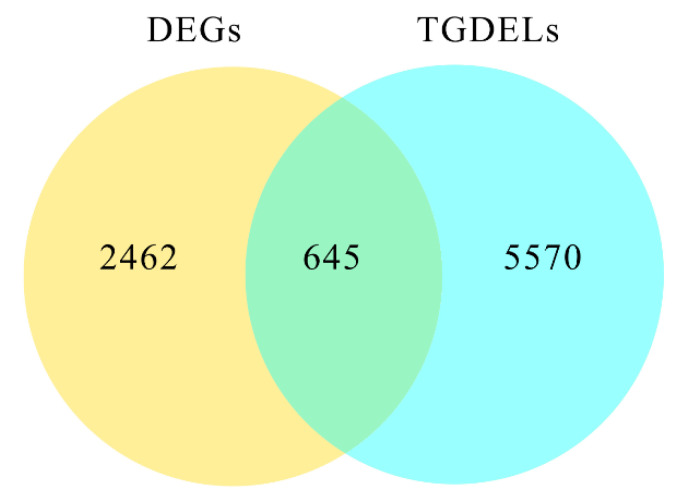
Venn diagrams of DEGTLs. DEGs: differentially expressed genes; TGDELs: target genes of DELs. Overlapping sections indicate these DEGs were predicted as DEGTLs.

In total, 6215 genes were predicted to be target genes of DELs (TGDELs) (Figure 5). Of these, 645 predicted target genes were also identified as DEGs (DEGTLs), suggesting that their differential expression might result from the differential expression of lncRNAs. In the 1 W vs. CK comparison, 376 DEGs were predicted to be targeted by 284 DELs; 273 DEGs were predicted to be target genes of 186 DELs in the 2 W vs. CK comparison; and 387 target genes of 269 DELs in the 3 W vs. CK comparison were found to be differentially expressed (Table S7).

A total of 117 common DELs and 144 common DEGTLs were found between the 1 W vs. CK and 2 W vs. CK comparisons, 137 common DELs and 167 common DEGTLs were found between the 1 W vs. CK and 3 W vs. CK comparisons, 134 common DELs and 191 common DEGTLs were found between the 2 W vs. CK and 3 W vs. CK comparisons, and 95 common DELs and 122 DEGTLs were found in all three comparisons (Table S8). Notably, TCONS_00063310, TCONS_00078175 and TCONS_00029828 were co-differentially expressed in kiwifruit during low-temperature storage. TCONS_00063310 and its target gene Acc21588 (pectinesterase, *PE*), TCONS_00078175 and its target gene Acc28966 (*BAM*), TCONS_00029828 and its target gene Acc17513 (Cytochrome b561 and DOMON domain-containing protein) were significantly upregulated throughout the storage stages. In addition, some DELs and their target *TF* genes showed common significant differential expression in three comparisons. For example, TCONS_00019524 and its target gene Acc14179 (*ZFP*), and TCONS_00091854 and its target gene Acc32998 (*NAC*), were significantly upregulated during low-temperature storage.

To reveal the potential regulatory roles of lncRNAs in kiwifruit during low-temperature storage, GO and KEGG enrichment analyses of DEGTLs were performed (Table S9 and S10). Compared with the CK group, 110, 62, and 91 GO terms were significantly enriched in the 1 W, 2 W, and 3 W groups, respectively. In addition, the DEGTLs identified in the three groups were all found to be significantly enriched in six GO terms, including ’transmembrane transport’, ‘transferase activity, transferring acyl groups’, ‘iron ion binding’, ‘transferase activity, transferring glycosyl groups’, ‘chlorophyll binding’, and ‘transferase activity, transferring acyl groups other than amino-acyl groups’. In addition, the ‘polysaccharide metabolic process’, ‘cell wall modification’, ‘glucosyltransferase activity’, ’pectinesterase activity’, and some other GO terms were significantly enriched in both comparisons of 1 W vs. CK and 3 W vs. CK.

**Figure 6:**
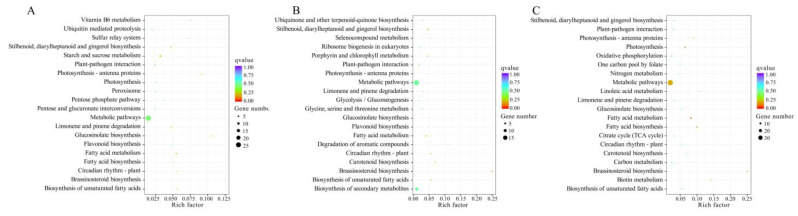
KEGG pathway enrichment analysis of DEGTLs in low-temperature stored kiwifruit. (**A**) Top 20 pathways enriched by DEGTLs in the comparison of 1 W vs. CK. (**B**) Top 20 pathways enriched by DEGTLs in the comparison of 2 W vs. CK. (**C**) Top 20 pathways enriched by DEGTLs in the comparison of 3 W vs. CK.

KEGG enrichment analysis showed that there were respectively 47, 40, and 52 pathways enriched by DEGTLs in kiwifruit after storage at 4 °C for 1, 2, and 3 weeks (Figure 6). Compared with the CK group, six pathways were significantly enriched (*p* < 0.05) in 1 W vs. CK comparison, including ’Fatty acid metabolism’, ‘Starch and sucrose metabolism’, ‘Glucosinolate biosynthesis’, ‘Photosynthesis-antenna proteins’, ‘Limonene and pinene degradation’, and ‘Stilbenoid, diarylheptanoid and gingerol biosynthesis’. After low-temperature storage for 2 W, six pathways were identified to be significantly enriched, including ‘Brassinosteroid biosynthesis’, ’Stilbenoid, diarylheptanoid and gingerol biosynthesis’, ‘Fatty acid metabolism’, ‘Carotenoid biosynthesis’, ‘Circadian rhythm-plant’, and ‘Biosynthesis of unsaturated fatty acids’. In the 3 W vs. CK comparison, seven pathways were significantly enriched, including ‘Fatty acid metabolism’, ‘Fatty acid biosynthesis’, ‘Photosynthesis’, ‘Brassinosteroid biosynthesis’, ‘Metabolic pathways’, ‘Biotin metabolism’ and ‘Photosynthesis-antenna proteins’. Among these pathways, ‘Fatty acid metabolism’ was identified to be significantly enriched in all three comparisons. Additionally, the ‘Starch and sucrose metabolism’, ‘Metabolic pathways’, ‘Plant-pathogen interaction’, and ‘Biosynthesis of secondary metabolites’ pathways were enriched by DEGTLs in all three comparisons.

### 2.7. QRT-PCR Verification

**Figure 7:**
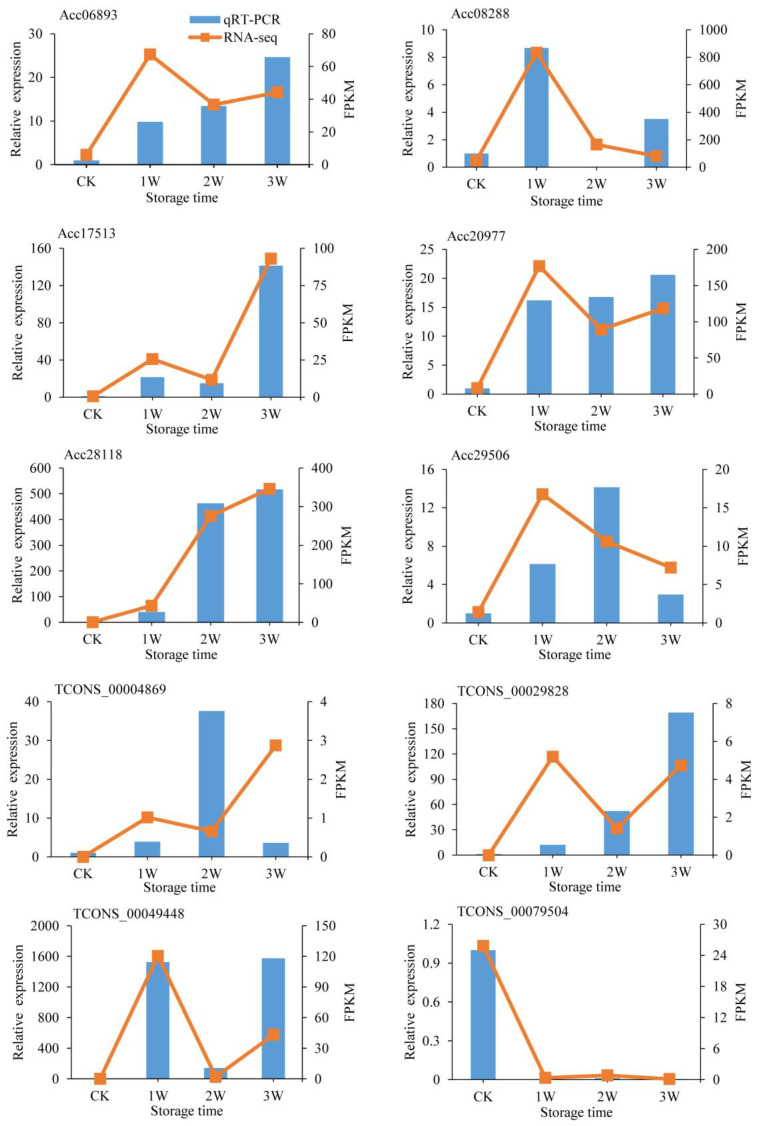
Expression analysis of selected genes and lncRNAs using qRT-PCR. The qRT-PCR results are shown in columns and the lncRNA-seq results are shown as curves. Different letters above columns represent a significant difference at *p*-value <0.05 level. CK, 1 W, 2 W, and 3 W indicate that kiwifruit stored at 4 °C for 0, 1, 2, and 3 weeks, respectively. Acc28118: Beta-amylase, Acc06893: Zinc finger protein, Acc20977: Beta-amyrin, Acc17513: Cytochrome b561 and DOMON domain-containing protein, Acc08288: *NAC*, Acc29506: Zinc transporter.

To verify the expressions of DEGs and DELs identified by lncRNA-seq data, six DEGs and four lncRNAs were subjected to qRT-PCR analysis (Figure 7). According to our lncRNA-seq results, of those transcripts, three genes (Acc28118 (*BAM*), Acc06893 (*ZFP*), and Acc20977 (Beta-amyrin)) and a lncRNA–target gene pair (TCONS_00029828 and Acc17513 (Cytochrome b561 and DOMON domain-containing protein)) were consistently upregulated after low temperature storage; one lncRNA (TCONS_00079504) was continuously downregulated after low-temperature storage. In addition, Acc29506 (Zinc transporter) and TCONS_00004869 were upregulated in 2 W and downregulated in 3 W groups. Furthermore, the expression of Acc08288 (*NAC*) and TCONS_00049448 showed a ‘rise-fall-rise’ change pattern during low-temperature storage. QRT-PCR analysis results showed that the expression trends of these selected transcripts were mostly consistent with our lncRNA-seq data, indicating that our lncRNA-seq data were believable.

## 3. Discussion

### 3.1. Cell Wall Metabolism Changes Contribute Greatly to the Fruit Softening of Kiwifruit during Low-Temperature Storage

Firmness change is one of the important characteristics during fruit ripening, and the metabolic action of the cell wall determines the rate of change in fruit firmness during ripening [42]. During fruit ripening, the cell wall structure undergoes a series of changes, including cellulose depolymerization, pectin degradation, and reduced intercellular adhesion [43]. During this process, enzymes associated with cell wall metabolism, such as β-galactosidase, xyloglucanendotransglycosylas (XET), endo-β-1,4-D-glucanase, pectinmethylesterase (PE), and polygalacturonase (PG), play indispensable roles. Endo-β-1,4-D-glucanase [44] and XET [45] are involved in the degradation of cellulose in the cell wall and promote the softening of the fruit cell wall. PE induces pectin degradation [46], which in turn reduces the mechanical properties of the fruit cell wall structure and effectively accelerates the rate of fruit softening. Degradation of pectin polymers in kiwifruit fruit can lead to increased cell gaps and loosening of cell wall structures, resulting in ripening and softening of kiwifruit [47]. In this study, the low-temperature storage of kiwifruit was found to be accompanied by decreased firmness but increased SSC, which are consistent with previous studies [40,48]. The pectinesterase activity GO term was significantly enriched by both DEGs and DEGTLs. Xyloglucan: xyloglucosyl transferase activity and glucan metabolic process related GO terms were significantly enriched by DEGs. In addition, the ascorbic acid content in kiwifruit is significantly reduced at the early stage of low-temperature storage. TCONS_00029828 targeted the gene Acc17513 (Cytochrome b561 and DOMON domain-containing protein) [49], which was reported to be involved in ascorbate regeneration, and was consistently upregulated. Meanwhile, the polysaccharide catabolic process GO term was significantly enriched by both DEGs and DEGTLs. Ascorbic acid facilitates oxidative splitting of polysaccharides in the plant cell wall and negatively regulates fruit firmness [50]. These results indicate that the cell wall metabolism changes contribute greatly to the fruit softening of kiwifruit during low-temperature storage.

### 3.2. LncRNAs Might Play Roles in Regulating Kiwifruit Ripening and Softening during Low-Temperature Storage by Mediating the Expression of Some TF Genes

Recently, lncRNAs have been found to play a vital role in plant responses to low temperature [51]. In this study, 3107 DEGs and 591 DELs were identified, and there were remarkably more upregulated DEGs and DELs than downregulated ones during low-temperature storage. TFs can activate or inhibit target gene expression by identifying functional elements on downstream gene promoters, thereby causing changes in plant adaptability to external stress stimulation [52]. In this study, several target *TF* genes (such as *NAC* and *ZFP*) of DELs also showed significant differential expression in kiwifruit during low-temperature storage. The genome-wide analysis of kiwifruit revealed that 125 of 142 *NAC* genes could be detected in fruit samples, and, among these, *AeNAC054*, *AeNAC061*, and *AeNAC118* showed high expression levels during fruit development [53]. Meanwhile, a regulatory pathway was found in ripening kiwifruit for ZFP (AdDof3) and *BAM* (*AdBAM3L*), and AdDof3 was found to contribute to kiwifruit ripening via binding and activation on the promoter of *AdBAM3L* [27]. Evidently, lncRNAs play crucial roles in kiwifruit’s response to low-temperature storage by regulating the expression of some *TF* genes.

### 3.3. LncRNAs Greatly Influence Kiwifruit Ripening and Softening during Low-Temperature Storage by Regulating Starch and Sucrose Metabolism and Cell Wall Modification

LncRNA-seq technology can accurately detect mRNA and non-coding RNA changes in samples and has been successfully used in studying biological functions of lncRNAs in ripening and softening of fleshy fruits, such as *S. lycopersicum* [54,55], *H. rhamnoides* [56], and *F. ananassa* [57]. In this study, GO enrichment analysis showed that the differentially expressed target genes of DEGTLs were significantly enriched in the pectinesterase activity and cell wall modification in both comparisons of CK vs. 1 W and CK vs. 3 W, which is consistent with the previous finding that the expression of cell wall modification related genes is induced in kiwifruit during low-temperature storage [13]. Fruit ripening and softening are mainly caused by cell wall hydrolysis, and this hydrolysis is a result of the coordinated action of cell-wall-modifying enzymes, including cellulase, PME, and PG. In *Capsicum frutescen*, PME enhances the rapid PG-mediated pectin degradation, leading to cell wall lysis [58]. In the presence of exogenous ethylene, several *PG* and *PME* genes are involved in polyuronide solubilization in the cell wall of *Prunus persica*, leading to decreased fruit hardness [59]. In addition, PG is involved in fruit ripening and softening of *Pyrus communis* [60], *Malus* × *domestica* Borkh [61], and *F. ananassa* [62]. Consistently, similar results were also found in our present study. The KEGG enrichment analyses of DEGs and DEGTLs both revealed that starch and sucrose metabolism is enriched in kiwifruit during low-temperature storage. In addition, two lncRNA-target gene pairs, i.e., TCONS_00063310-PE and TCONS_00078175-BAM, were predicted to play a key role in the starch and sucrose metabolism changes. Increased amylase activity contributes to fruit softening in kiwifruit [63], and low-temperature conditions result in the degradation of starch to soluble sugars; of these, BAM is one of the key enzymes involved in starch and sucrose metabolism during kiwifruit ripening [63]. In addition, *BAM* is considered to be a cold-responsive gene with a CRT/DRE element in its promoter that can be recognized by CBF. Therefore, CBF can directly regulate the content of sugar metabolites in low temperature stress by regulating the expression of *BAM* [20]. It is also reported that the AaCBF4-AaBAM3.1 module is involved in the transduction network related to kiwifruit freezing resistance [64]. Therefore, it can be concluded that lncRNAs play a major regulatory role in kiwifruit ripening and softening during low temperature storage by affecting starch and sucrose metabolism and cell wall modification.

## 4. Materials and Methods

### 4.1. Plant Materials

A. *chinensis* var. *deliciosa* cv. ‘Miliang 1’ fruits were harvested from the kiwifruit cultivation base of Fruit Research Institute, Fujian Academy of Agricultural Sciences, China (117°09′55.77″ E, 26°23′26.93″ N). At about 160 days after flowering, similar-sized kiwifruit without wounding and disease infection were harvested from 10 plants and stored at 4 °C for 0, 1, 2, and 3 weeks (herein named as CK, 1 W, 2 W, and 3 W groups, respectively). After treatment, fruits were collected, instantly frozen in liquid nitrogen, and stored in a freezer at −80 °C until further use.

### 4.2. Determination of Fruit Quality Parameters

The firmness and SSC of kiwifruit from different groups were measured using a hardness tester GY-4 (Top Instrument Co., Ltd., Zhejiang, China) and handheld Abbe refractometer (Atago, Tokyo, Japan), respectively. The vitamin C (Vc) contents of kiwifruit were determined using the 2,6-dichlorophenol indophenol method [65].

### 4.3. RNA Sequencing

#### 4.3.1. RNA Isolation

Total RNA of each sample was extracted using the RNAprep pure Plant Kit (Tiangen, Beijing, China), and its quality was firstly detected using 1.0% agarose gel electrophoresis. Then, a NanoPhotometer^®^ spectrophotometer (Implen, Los Angeles, CA, USA), a Qubit^®^ RNA Assay Kit in Qubit^®^ 2.0 Flurometer (Life Technologies, Carlsbad, CA, USA), and an RNA Nano 6000 Assay Kit of the Bioanalyzer 2100 system (Agilent Technologies, Santa Clara, CA, USA) were used to detect its purity, concentration, and integrity, respectively.

#### 4.3.2. Library Preparation and Sequencing

In total, 3 μg RNA was used as the input material of each sample. First, rRNA was removed using the Epicentre Ribo-zero™ rRNA Removal Kit (Epicentre, Madison, WI, USA). Then, the rRNA-free residue was cleaned through ethanol precipitation. Finally, the NEBNext^®^ Ultra™ Directional RNA Library Prep Kit for Illumina^®^ (NEB, Ipswich, MA, USA) was used to generate sequencing libraries with the rRNA-depleted RNA. RNA fragmentation was performed using divalent cations at a high temperature in 5× NEBNext First-Strand Synthesis Reaction Buffer, and dTTPs of dNTPs in the reaction buffer were replaced with dUTPs. First-strand cDNA was synthesized using a random hexamer primer and M-MuLV Reverse Transcriptase (RNaseH), and second-strand cDNA was subsequently synthesized using DNA polymerase I and RNase H. Remaining overhangs were converted into blunt ends using exonuclease/polymerase. The library fragments were purified using the AMPure XPsystem (Beckman Coulter, Beverly, MA, USA), and library quality was determined using the Agilent Bioanalyzer 2100 system.

Index-coded samples were clustered on a cBot Cluster Generation System using TruSeq PE Cluster Kit v3-cBot-HS (Illumina, San Diego, CA, USA). Then, the libraries were sequenced on an Illumina Hiseq 2000 platform to generate 100 bp paired-end reads. To obtain clean data, adapter-containing, poly-N-containing, and low-quality reads were removed from raw data through in-house Perl scripts. Simultaneously, Q20, Q30, and GC contents of the clean data were calculated. The lncRNA-seq clean data for kiwifruit stored at 4 °C for 0, 1, 2, and 3 weeks has been deposited in the Sequence Read Archive of National Center for Biotechnology Information database (NCBI, https://www.ncbi.nlm.nih.gov/sra/, accessed on 20 October 2022) under the accession numbers of SRX8554170, SRX8554171, SRX8554172, and SRX8554173, respectively.

#### 4.3.3. Read Mapping and Transcriptome Assembling

The kiwifruit reference genome and its gene model annotation files [66] were downloaded from NCBI. The index of the reference genome was built using Bowtie v2.0.6, and paired-end clean reads were aligned to this reference genome using TopHat v2.0.9. The mapped reads of each sample were assembled using both Scripture (beta2) [67] and Cufflinks (v2.1.1) [68] in a reference-based approach. The scripture was run with default parameters, and Cufflinks was run with ‘min-frags-per-transfrag = 0’ and ‘-library-type’; other parameters were set as default.

#### 4.3.4. LncRNA and MRNA Identification

For the screening of candidate non-coding RNAs, the coding potential calculator and pfamscan were applied. Of these, non-coding transcripts longer than 200 bp were selected as lncRNAs, which were further classified into several categories according to their genomic location and previous description. The coding genes located within 10 k/100 k upstream and downstream of an lncRNA were predicted as its *cis* target genes. Cuffdiff (v2.1.1) was used for the normalization of expression level of both lncRNAs and PCGs in each sample to fragments per kilobase million (FPKM) [68]. The Pearson’s correlation of the expression levels of an lncRNA and coding gene were also calculated, and the PCGs sharing absolute correlation values > 0.95 with a lncRNA were predicted as its *trans* target genes. The DEGseq package was used to identify DELs and differentially expressed genes (DEGs) with corrected *p*-value < 0.05 and |log_2_^FC^| ≥ 2.0 as criteria.

#### 4.3.5. GO and KEGG Enrichment Analyses

The GOseq R package was used for gene ontology (GO) enrichment analysis of DEGs or DEGTLs [69]. KOBAS software was applied to test the statistical enrichment of DEGs or DEGTLs in KEGG pathways [70]. GO terms and KEGG pathways with corrected *p* < 0.05 were considered as significantly enriched.

### 4.4. Quantitative Analysis of Real-Time Polymerase Chain Reaction

The same total RNA used for sequencing was reverse transcribed into cDNA using the PrimeScript™ RT reagent (Perfect Real Time) kit (TaKaRa, Shiga, Japan) for quantitative real-time polymerase chain reaction analysis (qRT-PCR), which was performed on a LightCycler 480 (Roche, Rotkreuz, CH) with *Actin* (Achn10718) as the internal reference gene. The relative expression levels of selected lncRNAs and genes were calculated using the 2^−∆∆Ct^ method. The information regarding primers used in this study are shown in Supplementary Materials Table S11.

## 5. Conclusions

In this study, we performed a dynamic lncRNA-seq analysis of kiwifruit during low-temperature storage. A total of 591 differentially expressed (DE) lncRNAs (DELs) and 3107 DE genes (DEGs) were identified, and 645 DEGs were predicted to be targets of DELs (DEGTLs), including some DE protein-coding genes (e.g., *BAM* and *PE*) and DE *TFs*. Combining the results of physical properties, and GO and KEGG enrichment analyses of DEGs, we found that the cell wall metabolism changes contribute greatly to the fruit softening of kiwifruit during low-temperature storage. Meanwhile, DEGTL-based GO and KEGG enrichment analyses revealed that lncRNAs influenced the ripening and softening of kiwifruit at low temperatures by regulating the expression of *TF* genes such as *NAC* and *ZFP*. Furthermore, lncRNAs play a major regulatory role in kiwifruit ripening and softening during low-temperature storage, mainly by regulating the genes related to the starch and sucrose metabolism and cell wall modification pathways.

## Figures and Tables

**Table 1:** Quality analysis result of the lncRNA-seq data for kiwifruit stored at 4 °C for different times. CK, 1 W, 2 W, and 3 W represent kiwifruit stored at 4 °C for 0, 1, 2, and 3 weeks, respectively.

Samples	Clean Reads	Q_20_ (%)	Q_30_ (%)	GC Content (%)	Total Mapped Reads	Multiple Mapped Reads	Uniquely Mapped Reads
CK	96,299,434	97.41	93.39	49.70	72,345,748 (75.13%)	4,550,388 (4.73%)	67,795,360 (70.40%)
1 W	95,968,050	96.76	91.99	45.40	75,104,959 (78.26%)	3,731,187 (3.89%)	71,373,772 (74.37%)
2 W	102,409,444	96.57	91.56	48.78	77,100,917 (75.29%)	6,090,122 (5.95%)	71,010,795 (69.34%)
3 W	127,966,632	96.79	92.05	47.77	97,768,730 (76.40%)	4,571,333 (3.57%)	93,197,397 (72.83%)

## Data Availability

The data supporting reported results can be found at https://kiwifruitgenome.org/ (accessed on 20 October 2022) and Supplementary Materials.

## Supplementary Materials

The following supporting information can be downloaded at: https://www.mdpi.com/article/10.3390/plants12051070/s1; Table S1: FPKM values of DEGs in kiwifruit stored at low temperature for different times; Table S2: FPKM values of commonly DEGs in kiwifruit stored at low temperature for different times; Table S3: FPKM values of DELs in kiwifruit stored at low temperature for different times; Table S4: FPKM values of commonly DELs in kiwifruit stored at low temperature for different times; Table S5: GO enrichment analysis results based on DEGs identified in this study; Table S6: KEGG enrichment analysis results based on DEGs identified in this study; Table S7: The kiwifruit DELs with differentially expressed target genes during low-temperature storage; Table S8: Common DELs and DEGTLs in each comparison group; Table S9: GO enrichment analysis results based on DEGTLs identified in this study; Table S10: KEGG enrichment analysis results based on DEGTLs identified in this study; Table S11: List of qRT-PCR primers used in the study.
