# Supplementary material for: Identification and Characterization of Long Non-Coding RNAs: Implicating Insights into Their Regulatory Role in Kiwifruit Ripening and Softening during Low-Temperature Storage
Source: Plants (Basel). 2023 Feb 27;12(5):1070. doi: 10.3390/plants12051070 (PMC10005093; doi:10.3390/plants12051070)
Supplement: Supplementary file 1 [file plants-12-01070-s001.zip › Table S11. List of qRT-PCR primers used in the study.pdf]

Table S11 List of qRT-PCR primers used in the study

**Gene_ID**	**Primer sequence (5′-3′)**		**Description**
	**Upstream**	**Downstream**	
Acc29506	GTGGAAGTGAAGGAGAGGAATG	CGAATGGTACACGGGCTATTT	Zinc transporter
Acc17513	ACGATGTCTCTGGTGCTAATG	CAGGGTCCGCAAATTCAAATAC	Cytochrome b561 and DOMON domain-containing protein
Acc08288	CGTTGTGAAGAATGGTGTCATAAG	ATTCCAGTGGCCTTCCAATAG	NAC
Acc20977	AGGAGCTTGTGAGTGTGATAAA	TCTGATAGGACCCTGTTGTAGA	Beta-amyrin
Acc18497	CGATAGAGTCTCCCTTGCTTTAAT	CAGTACCATCTCACGCAACTC	Late embryogenesis abundant protein
Acc28118	CATGACTCCGGCCAGTATAAC	CCGAGTACCACTCTAGGAAGAA	Beta-amylase
Acc06893	ATGGCACTTGAAGCACTCA	GTAGGACGGGAAGGATTTGTC	Zinc finger protein
TCONS_00029828	GAGGGCTAAGAAGAGGCACA	TGGGTTTCAGTCGTTGTATCTC	Long non-coding RNA
TCONS_00049448	TTCTTGCCTTCGCCTTCT	AATGTCTTTGCCCTTCCG	Long non-coding RNA
TCONS_00079504	TGTGGAGGAAGAAGAAGAAGAAG	ACTCGGTAAGTGGTGAAACAG	Long non-coding RNA
TCONS_00004869	TGGGAAACTCACTCAAACTCC	TTGGCAAGGCTATTCAGAAG	Long non-coding RNA
